# QCM-based assay designs for human serum albumin

**DOI:** 10.1007/s00216-021-03771-0

**Published:** 2021-12-23

**Authors:** Wisnu Arfian A. Sudjarwo, Mathias Thomas Dobler, Peter A. Lieberzeit

**Affiliations:** University of Vienna, Faculty for Chemistry, Department of Physical Chemistry, Waehringer Strasse 42, 1090 Vienna, Austria

**Keywords:** Molecularly imprinted polymer, Nanoparticles, Competitive assay, Human serum albumin, Quartz crystal microbalance

## Abstract

Solid-phase synthesis is an elegant way to create molecularly imprinted polymer nanoparticles (nano-MIPs) comprising a single binding site, i.e. mimics of antibodies. When using human serum albumin (HSA) as the template, one achieves nano-MIPs with 53 ± 19 nm diameter, while non-imprinted polymer nanoparticles (nano-NIPs) reach 191 ± 96 nm. Fluorescence assays lead to Stern–Volmer plots revealing selective binding to HSA with selectivity factors of 1.2 compared to bovine serum albumin (BSA), 1.9 for lysozyme, and 4.1 for pepsin. Direct quartz crystal microbalance (QCM) assays confirm these results: nano-MIPs bind to HSA immobilized on QCM surfaces. This opens the way for competitive QCM-based assays for HSA: adding HSA to nanoparticle solutions indeed reduces binding to the QCM surfaces in a concentration-dependent manner. They achieve a limit of detection (LoD) of 80 nM and a limit of quantification (LoQ) of 244 nM. Furthermore, the assay shows recovery rates around 100% for HSA even in the presence of competing analytes.

**Supplementary Information:**

The online version contains supplementary material available at 10.1007/s00216-021-03771-0.

## Introduction

Human serum albumin (HSA) contains 585 amino acids and is widely known as the most abundant protein in the human body, at concentrations of approximately 3.5–5 g/dL. It plays important roles in body metabolism including metabolite delivery, enzymatic activity, plasma oncotic pressure regulation, and antioxidant properties [1, 2]. Abnormal levels are indicative of health problems: increased HSA levels—so-called hyperalbuminemia—are usually associated with a protein-rich diet combined with a high level of exercise [3]. In the same way, decreased HSA levels are warning signals: for example, chronic liver diseases with hepatocyte malfunction reduce HSA synthesis. Therefore, the level of HSA is a marker of liver function; low HSA concentration—called hypoalbuminemia—is often a consequence of cirrhosis [2, 4]. Furthermore, decreasing HSA levels correlate with the prognosis of hepatocellular carcinoma and increasing tumor diameter [5, 6].

Thus, the detection of HSA levels is of fundamental interest. Current methods include immunoassays [7], electrochemical detection [8], liquid chromatography (LC) [9], fluorescence probes [10, 11], colorimetric assays [12], and sensing with field effect transistors (FET) [13, 14]. Nonetheless, these methods suffer from limitations such as cost-effectiveness, size of instrumentation, protocol complexity, and specific environments for electrochemical assays. Herein we report an assay based on quartz crystal microbalance (QCM) sensors that allow for straightforward measurements that do not require any complex procedures to pretreat the samples. QCMs are the most widely used mass-sensitive devices: their resonance frequency changes when mass deposits on the electrode surface. Of course, these devices are not selective per se and require the use of a suitable receptor, in this case a molecularly imprinted polymer (MIP).

MIPs are artificial recognition materials and template-based receptors with the ability to recognize defined target species via non-covalent interactions. Their synthesis relies on co-polymerization of monomer(s) and crosslinker in the presence of a template [15], which is usually the target species of the sensor. In the case of large templates, often biospecies including proteins and entire cells, the most widely applied approach is surface imprinting [16, 17]. Some recent QCM studies for detecting proteins with MIPs include targets such as bovine hemoglobin [18], dopaminergic D1 receptor [19], low-density lipoprotein [20], trypsin [21], heparin [22], and insulin [23]. A rather recent approach for synthesizing “monoclonal” MIPs against biological targets relies on solid-phase synthesis of so-called nano-MIPs [24]. The main idea behind this approach is to synthesize MIP nanoparticles through solid-phase synthesis with the template immobilized on a substrate. Two subsequent washing steps—one with cold solvent and one with warm—first remove low-affinity particles and then allow for eluting high-affinity ones. The resulting nano-MIPs are expected to comprise only one binding site per particle.

Herein, we investigate nano-MIPs for sensing of HSA in situ. For that purpose, we carried out both direct measurements on QCM surfaces and a competitive biomimetic “pseudo-immunoassay.” This makes it necessary to reverse the logic of the sensor measurement: instead of coating the MIP onto the electrode and exposing it to the analyte, we immobilized HSA—the target species—on the surface and used it to capture the (much larger) MIP particle. This approach of using a small probe for capturing a larger species is in line with our previous work on detecting DNA amplification products on QCM [25].

## Materials and methods

### Reagents

*N*-Isopropylacrylamide (NIPAm), *N*,*N*,*N*′,*N*′-tetramethylethylenediamine (TEMED), *N*-tert-butylacrylamide (TBAm), phosphate-buffered saline (PBS), and *N*,*N*′-methylenebisacrylamide (BIS) were purchased from Alfa Aesar (USA). Ammonium persulfate (APS) was purchased from Acros (USA), glutaraldehyde (GA) was produced by Amresco (USA), and silica gel, acrylic acid (AAc), 3-aminopropyltriethoxysilane (APTES), HSA, lysozyme, pepsin, bovine serum albumin (BSA), cysteamine, ethanolamine, ethanol, and toluene were obtained from Merck (Germany). Acetone was obtained from VWR Chemicals (USA), and *N*-(3-aminopropyl) methacrylamide hydrochloride (APM) was purchased from Polysciences, Inc. (USA).

### Preparing the matrix for solid-phase synthesis

To activate silica gels as support materials for MIP synthesis, we added 2 g silica gel to 4 mL 1 M NaOH solution and boiled for 15 min. Afterwards, silica gels were first washed with PBS (pH 7.4; 10 mM) and then by rinsing with excess of distilled water followed by excess of acetone at room temperature. They were then left to dry in the oven for 2 h at 80 °C. Finally, we added the product to a solution of APTES in toluene (2.5% *v*/v) and rotated the batch in a tube rotator (speed 20 rpm) overnight. Finally, we filtered and rinsed with a mixture of acetone/ethanol (4:1) and dried the filtrate in a vacuum funnel to obtain amino-functionalized silica gel.

After this, we added the product to a solution of glutaraldehyde (GA) in PBS (pH 7.4) (c = 7.5% *v*/v) for 2 h. Imine formation as a result of mixing APTES and GA caused the color to change from colorless to yellow or orange. We then rinsed the solid with distilled water and dried it under vacuum. To immobilize HSA, we incubated modified silica gels in 500 μg/mL of HSA solution (2 mL HSA solution per gram silica gel). The mixture was placed in a refrigerator overnight at 4 °C. Silica gels were then filtered and rinsed with PBS followed by water before drying at 4 °C. The final stage was to block free aldehyde groups with ethanolamine 50 mM for 15 min. Finally, we washed the silica gels with water and dried them at 4 °C.

### Synthesis of nano-MIPs

**Scheme 1:**
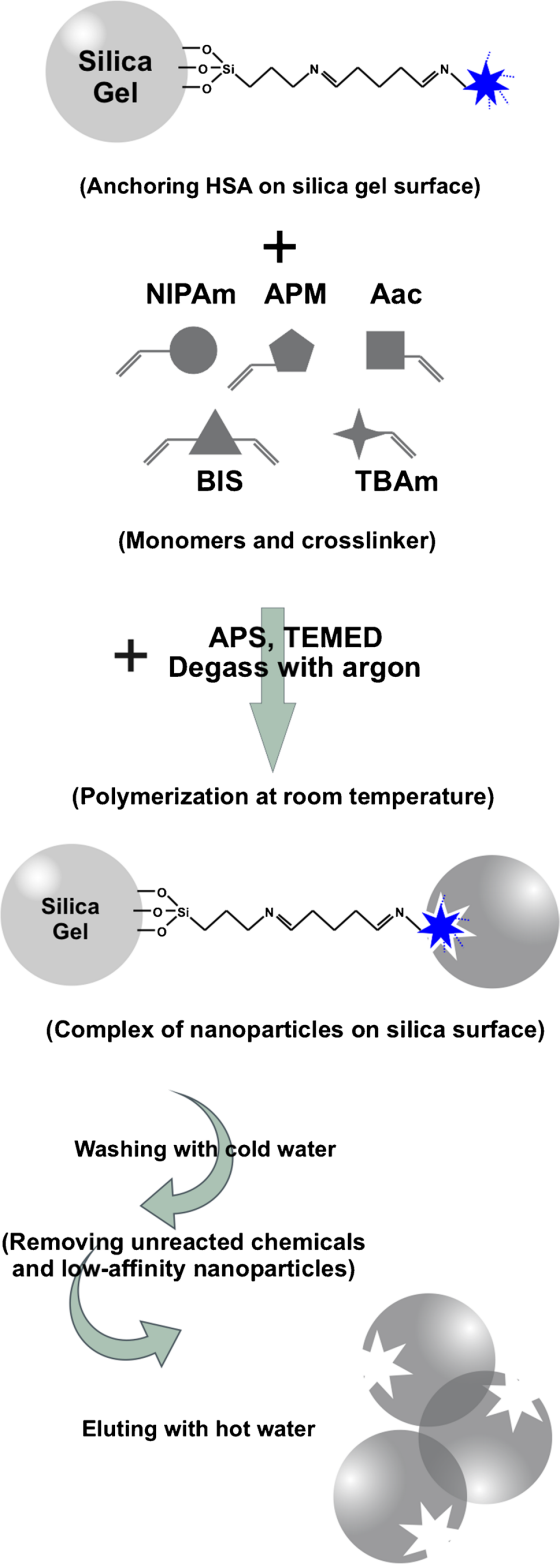
Solid-phase synthesis of MIP nanoparticles

Scheme 1 summarizes the steps for the preparation of nano-MIPs by adapting a procedure from Canfarotta et al. [26]: We added 50 mg NIPAm, 3.1 mg BIS, 43 mg TBAm, 15.1 μL AAc, and 6 mg APM to a mixture of 500 μL ethanol and 2500 μL water. The solution was then sonicated for 10 min followed by injecting 45 μL of 20% (m/v) APS solution and 45 μL of 20% (*v*/v) TEMED solution. Then, we added 1–2 g modified silica gel to the solution while purging the mixture with argon for 10 min. Thereafter, the mixture stayed at room temperature for 2 h to finish polymerization. Subsequently the mixture was filled into a filter column and eluted with water at 15 °C to remove unreacted chemicals and both low-affinity and non-imprinted nanoparticles. To harvest high-affinity nano-MIPs, we flushed the column with 30–50 mL water at 65 °C. As a reference, non-imprinted polymer nanoparticles (nano-NIPs) were synthesized following the same procedure, leaving out GA and HSA.

### Determining nano-MIP yield

Before utilizing nano-MIPs in any experiments, we evaporated the solvent from 2 mL of an aliquot of nano-MIP suspension and weighed out the mass of the solid residue to obtain the concentration of the suspension. The same method also served to determine the concentration of nano-NIPs. To increase particle concentrations prior to measurements, we evaporated the solvent from 10 mL solution at 60–65 °C until less than 1 mL was left, transferred to 1 mL Eppendorf tube, and added water to yield 1 mL. This stock solution was used to prepare all other samples for sensor measurements.

### Characterization of nano-MIPs

Three different measuring techniques were used to determine the size of the nano-MIPs, namely dynamic light scattering (DLS), scanning electron microscopy (SEM), and atomic force microscopy (AFM). About 2 mL of high-affinity nano-MIPs was filtered using a CHROMAFIL Polyethylene (PE)-20/25 (0.2 μm) syringe filter and sonicated for 10 min to homogenize the sample. It was then analyzed using DLS (Zetasizer Nano). For SEM and AFM measurements, about 100 μg/mL of nano-MIP solution was dropped on gold electrodes and dried at 80 °C for 1 h.

### Fluorescence characterization of affinity

Fluorescence spectroscopy was carried out to characterize the affinity binding of the nano-MIPs and nano-NIPs against selected proteins. For that purpose, we set the final concentration of HSA at 150 μg/mL after mixing with either nano-MIPs or nano-NIPs. The various concentrations of both nano-MIPs and nano-NIPs were 50, 100, 150, 200, 250, 300, 350 and 500 μg/mL. Fluorescence measurements used 278 nm for HSA.

### Sensing apparatus

To produce QCM, we referred to a previously published method by Latif et al. [27]. In brief, we screen-printed dual-electrode patterns onto the surface of 10 MHz AT-cut quartz slides (diameter 13.8 mm, purchased from Roditi Inc., UK) with 10% brilliant gold paste (Heraeus, Germany). After that, we cured at 400 °C for 4 h to remove organic residues and expose the bare gold. A network analyzer (Agilent 8712ET) was used to characterize QCM according to their respective resonance frequencies and damping values. AFM (Bruker instrument), DLS (Zetasizer Nano, Malvern Instruments Ltd.), SEM (Zeiss Supra 55VP), and transmission electron microscopy (Philips CM 200) were employed to measure the size of the nano-MIPs. Fluorescence spectroscopy (PerkinElmer LS 50B) was used to investigate binding affinity.

### Preparing QCM sensor surfaces

**Scheme 2:**
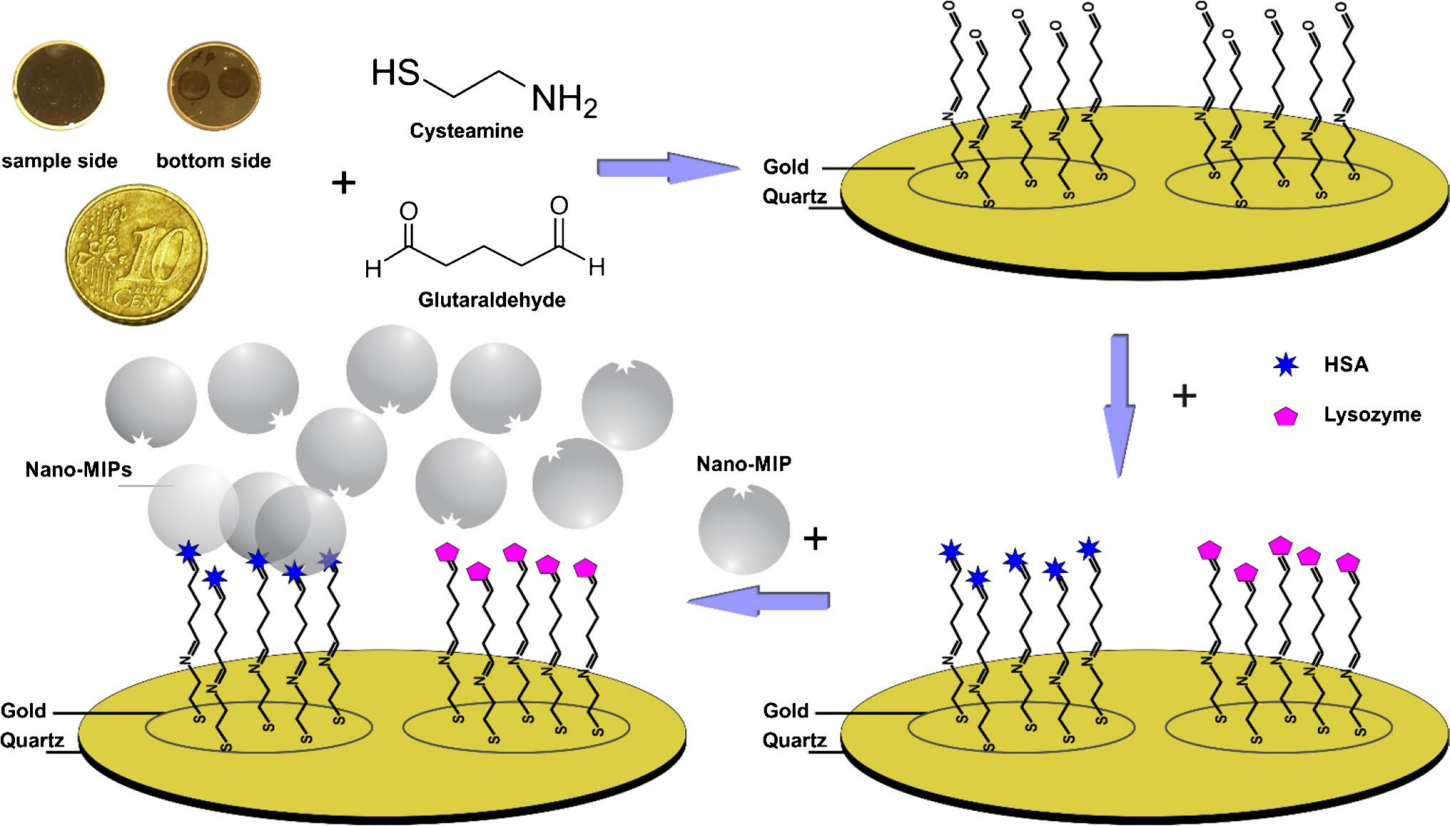
Sensor setup for QCM measurements

Scheme 2 summarizes the steps leading to the QCM-based assay format. Before starting, we cleaned the QCM gold electrode surfaces with acetone and alkaline piranha solution (NH_4_OH:H_2_O_2_:H_2_O = 1:1:5) by dropping it onto the surface and allowing it to react for 45 s. After washing with distilled water, the QCM surfaces were dried in a vacuum desiccator or flushed with air.

Then, we placed the clean QCM into 2.5 mM aqueous cysteamine solution overnight in a sealed dish stored in the dark to introduce amino functionalities. The sensors were then washed with Milli-Q water and dried at room temperature or by flushing them with argon gas. Afterwards, we immersed them in 5 mM aqueous GA solution for 1.5 h, followed by washing with water and drying in an argon gas stream. To modify the surface, we then injected 10 μl of 500 μg/mL HSA solution for immobilization into the measuring cell. After keeping in the refrigerator for 1.5 h, we rinsed the QCM with water and repeated the process a second time. The final step was to block the unreacted aldehyde groups using 0.50 mM aqueous solution of ethanolamine for 20 min. Thereupon chips were cleaned using Milli-Q water, dried using argon gas, and stored in the refrigerator until measurements. To ensure successful production of the assay, one electrode of each dual-electrode QCM was modified with HSA and the other with only linker consisting of cysteamine and GA. This assay was investigated by injecting HSA in a custom-made measuring cell. The other electrode was later modified with another protein as a competitor for selectivity testing.

### QCM sensing experiments

Each QCM chip was placed in a custom-made measuring cell (for details see Fig. S1 in the electronic supplementary material) and connected to a frequency counter followed by injecting a concentration series of nano-MIPs. We monitored and recorded the resulting frequency shifts of both electrodes. As each QCM contained two different proteins on each electrode, the approach allowed us to study both the sensitivity and selectivity of particles in one measurement. We carried out all measurements 3–4 times each.

### Competitive assay

For competitive assays, we mixed nano-MIPs at a concentration of 500 μg/mL with the same volumes of HSA solutions (42, 36, 27, 17.5, 9 μg/mL, respectively). The frequency shift produced by injecting the mixture then led to the respective sensor characteristic. In addition, we also mixed nano-MIPs with HSA and one of the selected competing proteins, i.e. BSA, lysozyme, pepsin, and insulin, to study both the selectivity and recovery rate.

### Real sample analysis

To assess the quality of the sensor, we exposed it to commercially available human serum (TH Geyer, Germany). The expected HSA concentration of such a sample is in the range of c(HSA) = 40–45 g/l. These values are about a factor of 3000–3500 higher than the operational range of the sensor. To ensure that the sensor indeed responded in a dynamic manner, the two samples were prepared as follows: 56.7 μl (dilution 1) and 63.1 μl (dilution 2) of serum were diluted to 100 ml with deionized water. Aliquots of these solutions were mixed with an equal volume of nanoparticle solution (500 μg/mL) and injected into the sensing cell.

## Result and discussion

### Nano-MIP synthesis and characterization

**Fig. 1:**
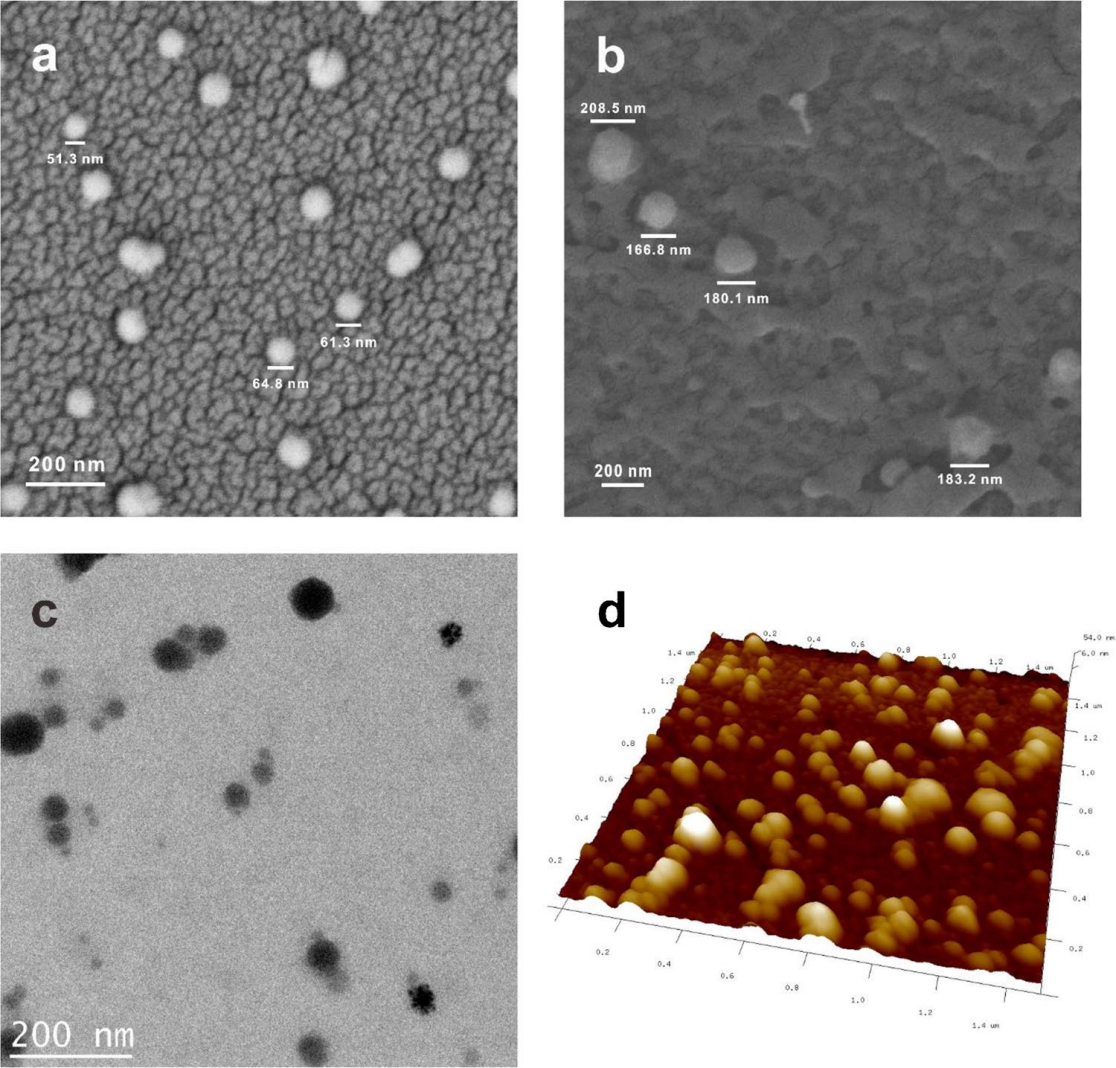
**a** SEM image of nano-MIPs, **b** SEM image of nano-NIPs, **c** TEM image of nano-MIPs, **d** AFM image of nano-MIPs. Vertical scale: 54.8 nm

The first important step in developing this assay is to confirm the efficacy of nano-MIP synthesis. Figure 1 shows SEM, AFM, and TEM images of the nanoparticles. The scale bar in each image denotes 200 nm. Obviously, the two SEM images (Fig. 1a, b) both show spherical particles after nano-MIP and nano-NIP synthesis, respectively. Their diameters are different, however, with nano-MIPs reaching around 50–60 nm, while nano-NIPs show around 200 nm. One can clearly discern the individual particles, as they do not aggregate. This is beneficial for sensing in situ, because one can expect each particle potentially being able to interact with an immobilized protein molecule. DLS results confirm the SEM images: the sizes of nano-MIPs and nano-NIPs are 53 ± 19 nm and 191 ± 96 nm, respectively, with polydispersity index PdI = 0.25 (see Fig. S2 in supplementary material). These wide standard deviations come from randomly oriented pores and networks in the silica gels. In the case of nano-NIPs, the absence of an immobilized template makes it even more difficult to properly control the polymerization process. Figure 1c shows a TEM image of nano-MIPs and Fig. 1d a respective AFM image. Both AFM and TEM images confirm the results including wide standard deviations.

Hence, the synthesis procedure proved feasible, because it allows for acquiring comparably high amounts of nano-MIPs. For instance, 1.5 g silica gel eluted with 50 mL water at 65 °C yields 250–450 mg/L particles.

### Affinity binding and interaction

**Fig. 2:**
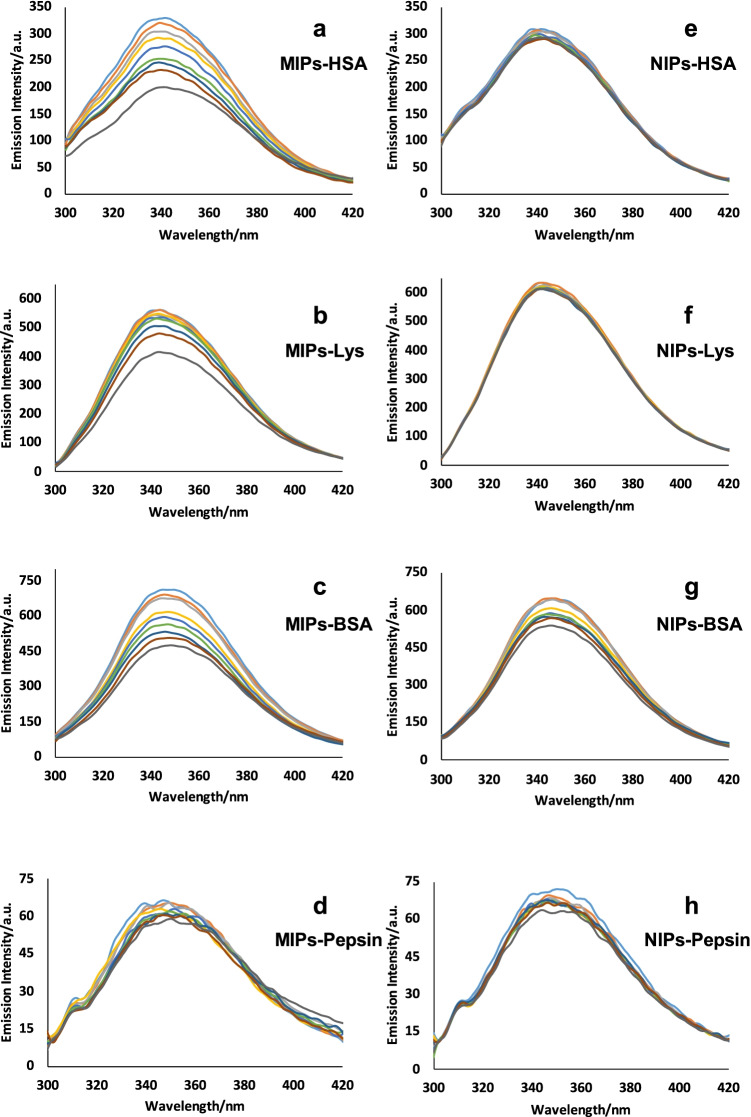
Fluorescence spectra obtained after interactions of **a** nano-MIPs–HSA, **b** nano-MIPs–lysozyme, **c** nano-MIPs–BSA, **d** nano-MIPs–pepsin, **e** nano-NIPs–HSA, **f** nano-NIPs–lysozyme, **g** nano-NIPs–BSA, **h** nano-NIPs–pepsin

Fluorescence spectroscopy offers a powerful tool to assess the binding affinity of nanoparticles to proteins: it is known that interactions between polymers and fluorescing species may lead to quenching [28–30]. Figure 2 shows series of fluorescence spectra of different proteins in the presence of nano-MIPs and nano-NIPs, respectively. Evidently, the different proteins show different amounts of quenching: The fluorescence spectra of HSA decrease in intensity when adding nano-MIPs to the solution. On the other hand, adding nano-NIPs hardly influences the fluorescence spectra, which means that hardly any non-specific interactions occur between nano-NIPs and HSA. Overall, nano-MIPs lead to 10 times higher Stern–Volmer constants K_SV_ than nano-NIPs, indicating ten times stronger binding (Table 1). The values for K_SV_ result from the slope of the respective Stern–Volmer plot. It is a measure for the affinity between nanoparticles and protein molecules, i.e. the larger K_sv_, the more affine the binding. Hence, nano-MIPs comprise a selective binding site toward HSA.

**Table 1:** Binding parameters of the interaction between nanoparticles and proteins acquired from fluorescence quenching

Type of Interaction	K_sv_ (L/mol) × 10^5^	R
Nano-MIPs–HSA	7.33	0.989
Nano-MIPs–BSA	6.00	0.994
Nano-MIPs–lysozyme	3.95	0.905
Nano-MIPs–pepsin	1.79	0.959
Nano-NIPs–HSA	0.71	0.912
Nano-NIPs–BSA	2.43	0.976
Nano-NIPs–lysozyme	0.39	0.903
Nano-NIPs–pepsin	0.73	0.912

The data shown in Fig. 2 and Table 1 also allow for assessing selectivity: for that purpose, we used the Stern–Volmer constants K_sv_ summarized in Table 1: interaction with HSA as an analyte leads to the largest value, followed by BSA. Although both proteins are rather similar in the number of amino acids (HSA = 585 amino acids; BSA = 583 amino acids) and their amino acid sequences, the conformational identity between them is around 71–76% [31]. Furthermore, HSA comprises only one tryptophan residue, while BSA has two. These tryptophan residues substantially affect fluorescence [32]. It is known that this plays an important role in binding [33]. This is also visible from the data: despite said similarities, K_SV_ for HSA is still more than 120% of that for BSA. It of course also shows that nano-MIPs lead to significant quenching in BSA solution. However, for all other selected proteins, the Stern–Volmer constants differ significantly from that of HSA, reaching factors of 1.85 and 4.09 for HSA-lysozyme and HSA-pepsin, respectively. Surprisingly, all constants produced by the interaction between nano-NIPs and the analyte proteins are small and generally negligible, with the exception of BSA. This further corroborates the selectivity of the MIP: BSA shows around 3.5 times higher non-specific binding to the polymer, than HSA.

### QCM assay

**Fig. 3:**
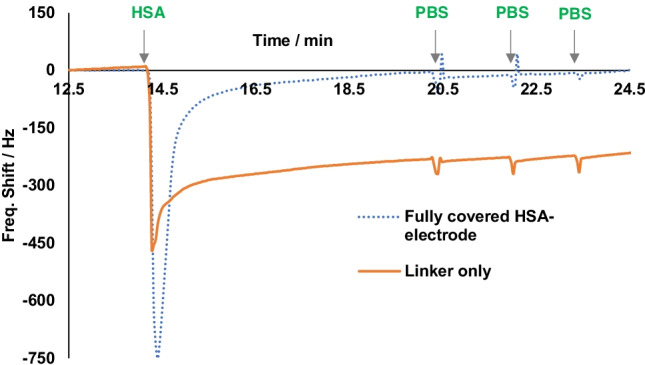
QCM data for two different self-assembled layers to test the immobilization of HSA and non-specific binding between protein molecules

Before using QCM prepared according to Scheme 2 for sensor measurements, it is imperative to ensure that it indeed contains the target protein anchored on the electrode surface. For that purpose, we exposed QCMs comprising free aldehyde groups on one electrode and immobilized HSA on the other to HSA solutions while recording the resonance frequencies of the device. Figure 3 shows the outcome: the blue dotted line represents the frequency shifts of the electrode fully covered with HSA. Evidently, they are reversible and negligible. This also clearly demonstrates that HSA does not aggregate from solution with HSA molecules immobilized on the sensor surface. This aspect is important not least for the competitive assay format. In contrast to this, the orange-colored line shows the frequency shifts of the electrode comprising free aldehyde groups (from immobilized glutaraldehyde) to the same sample injections. One can clearly see an irreversible frequency drop of −238 Hz (at electronic noise levels of 5 Hz), which is in line with the shifts that one would expect upon immobilizing a protein monolayer. The aldehyde groups obviously have reacted with the primary amino groups on the outside of the HSA molecules and thus immobilized them on the surface. The next step confirms this even further: even after flushing the measuring cell three times with PBS, the signals of both channels reverted back to their respective equilibrium positions. The results allow for two conclusions: First, the procedure is highly feasible for immobilizing HSA on QCM electrode surfaces. Second, there is only negligible interaction between HSA molecules, i.e. they do not tend to aggregate on the surface. One can observe the same patterns for other proteins due to the injection into aldehyde-modified QCM (Fig. S4 supplementary material).

### Direct sensing with QCM

**Fig. 4:**
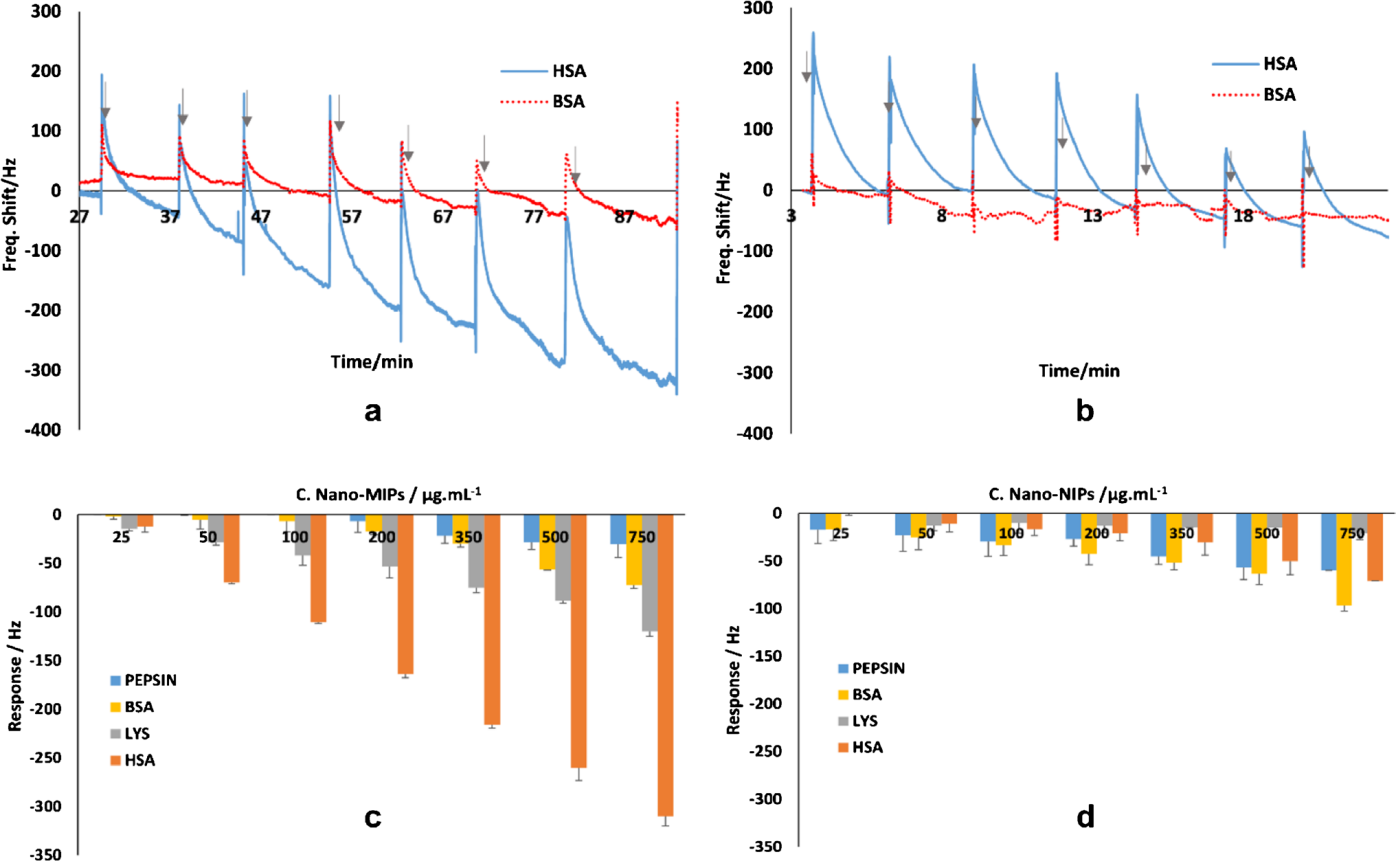
**a** QCM results of HSA- and BSA-modified electrodes exposed to nano-MIPs, **b** QCM results of HSA- and BSA-modified electrodes exposed to nano-NIPs, **c** the responses produced by nano-MIPs toward selected proteins, **d** the responses produced by nano-NIPs toward selected proteins

Figure 4a, b summarizes the sensor responses of the direct QCM assay, i.e. when exposing the sensors to nano-MIPs and nano-NIPs. It also shows the results of QCM measurement demonstrating the selectivity and sensitivity between HSA and BSA. When using nano-MIPs in a concentration range from 25 to 750 μg/mL HSA, the sensor yields frequency shifts from −44 to −324 Hz. In contrast, nano-NIPs lead to only −13 to −78 Hz for HSA as a result of non-specific interactions between the nanoparticles and protein molecules. In both cases, response times to achieve equilibrium signal were around 10 min, which is also appreciably rapid in the light of potential real-life application. The nano-MIPs are ~4.2 times more sensitive than nano-NIPs, which clearly demonstrates the role of their tailored interaction cavity for HSA recognition. The mass-sensitive results seem reasonable: when fully covering the electrode surface with a monolayer of MIP nanoparticles, one would expect frequency shifts below −800 Hz. Details about the calculation can be found in the electronic supplementary material.

Figure 4c, d shows the results of selectivity tests toward other proteins, namely BSA, lysozyme, and pepsin. Frequency changes due to nano-MIP injection are around −19 to −75 Hz for BSA, −13 to −120 Hz for lysozyme, and −15 to −50 Hz for pepsin. Injecting nano-NIPs in general generates low responses from −13 to −49 Hz for BSA, −5 to −30 Hz for lysozyme, and −5 to −70 Hz for pepsin. Triplicate measurements reveal standard deviations of 2–17 Hz. The selectivity factors of nano-MIPs are 2.7 for HSA-lysozyme, 4.5 for HSA-BSA, and 10.6 for HSA-pepsin, which are very appreciable results. The selective interactions of the nano-MIPs with the respective protein tend to correlate with the molecular size (molecular mass) and isoelectric point: the molar masses of selected proteins are 66.5 KDa, 66.5 KDa, 14.3 KDa, and 34.5 KDa for HSA, BSA, lysozyme, and pepsin, respectively. Meanwhile, the isoelectric points differ much more, with 4.9 for HSA [34], 4.7 for BSA [35], 10.7 for lysozyme [36], and 2.7 for pepsin [37]. Both parameters, indeed, affect how nano-MIPs capture the respective analyte: for instance, lysozyme at a pH of the buffer exhibits positive net charge and thus electrostatically supports binding of the protein to the polymer and vice versa. Changing the environment affects not only the net charge, but also the conformation of protein [38, 39], which of course influences binding. Nonetheless, when synthesis and measuring conditions are similar to each other, the imprinting process leads to cavities corresponding to template size and conformation in the polymer. In contrast to this, nano-NIPs reveal only minor sensitivity for all selected proteins. These results also correlate well with the fluorescence assay, which is selective toward HSA.

### QCM-based competitive assay

**Fig. 5:**
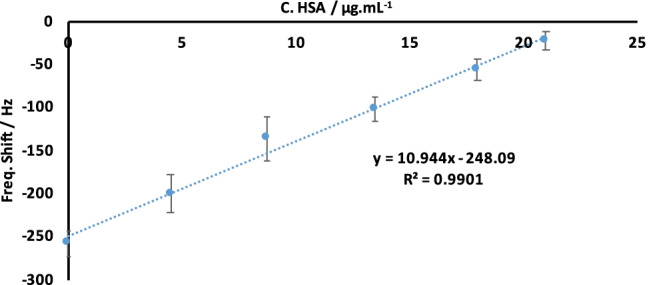
QCM sensor characteristic in a competitive assay toward HSA

Figure 5 presents the sensor characteristic of QCM-based competitive assay based on injecting mixed solutions of nano-MIPs and HSA. One can clearly see that the QCM sensor responses decrease with increasing HSA concentration in solution, which is in line with competitive assays: only unbound nano-MIP particles can actually interact with an HSA molecule on the quartz surface. Adding low concentrations of HSA leads to comparably high frequency shifts on the sensor and high concentrations to small frequency shifts. For instance, the concentration of 42 ppm HSA in the mixture led to effects of −20 Hz, while 9 ppm of HSA produced a frequency change up to −205 Hz.

We calculated the limit of detection (LoD) as 3.3 × (standard deviation of the intercept)/slope and the limit of quantification LoQ as 10 × (standard deviation of the intercept)/slope [40]. The standard deviation of this sensor was 17.7 Hz and the slope was 10.944, resulting in LoD values of 5.3 μg/mL (80 nM) and LoQ of 16.2 μg/mL (244 nM). These data rely on triplicate measurements for each competitive assay on the same quartz. Between two measurements, one needs to regenerate the quartz by flushing it with warm water at T = 38–40 °C, which removes the bound nanoparticles from the surface.

**Fig. 6:**
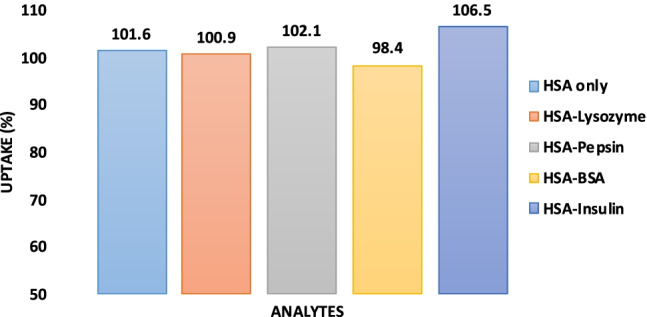
The percentage of HSA taken up by nano-MIPs with competitive assay

Finally, every sensor needs to demonstrate selectivity. In a first step, it is important to ensure that all proteins are actually immobilized on the respective QCM surface. This is indeed the case for the competitors chosen, namely BSA, lysozyme, and pepsin. As Fig. S4 (supplementary material) shows, exposing aldehyde-functionalized QCM electrodes to each of those compounds, respectively, gives rise to irreversible frequency shifts in the range of −50 to −70 Hz, indicating successful covalent binding. Figure 6 shows the outcome of the selectivity study in the competitive assay when mixing the nanoparticles with solutions containing the same concentrations of HSA and a competitor. The recovery rate of the assay for pure HSA is 101.6%, whereas in the cases of binary mixtures it is slightly different, namely 100.9%, 102.1%, 98.4%, and 106.5% for HSA–lysozyme, HSA–pepsin, HSA–BSA, and HSA–insulin, respectively. At a confidence level of 99%, the assay leads to a confidence interval in the range of 97.4–106.6%, which means that the differences between the aforementioned recovery rates are statistically nonsignificant. Hence, the overall recovery with the method is in the range of 98.4–106.5%, which is a highly appreciable figure.

In order to investigate the performance of the sensor in a real-life setting, we tested commercial human serum with the competitive assay developed herein and compared the results with those of clinical routine analysis at the central diagnostic laboratory of the Vienna General Hospital (results received after the QCM measurements had taken place). Table 2 summarizes the frequency shifts for two measurements of two different dilutions each (to test whether the sensor responded dynamically). On average, adding dilution 1 to the sample system led to frequency shifts of −95 Hz, and dilution 2 to shifts of −77 Hz. Inserting these values into the sensor characteristic shown in Fig. 5 leads to the HSA concentrations shown in Table 1. Overall this leads to c(HSA) = 50 ± 5 g/l from the sensor responses. Standard clinical analysis revealed c(HSA) = 52.5 g/l. Hence, the sensor result achieves slightly more than 94% of this standard method. Despite the complexity of the sample matrix, this is a highly appreciable outcome, even though nanoparticles seem to interact not only with HSA, but to a minor extent also with other proteins in the serum.

**Table 2:** Outcome of sensing real serum samples

Sample	Dilution 1 (1:3530)	Dilution 2 (1:3170)
Measurement 1	−95 Hz	−77 Hz
Measurement 2	−94 Hz	−78 Hz
c(diluted sample)	14 ± 1 mg/l	16 ± 2 mg/l
c(original serum)	49 ± 5 g/l	50 ± 5 g/l

## Conclusion

With the advent of MIP nanoparticles resulting from solid-phase synthesis, molecular imprinting has come closer to delivering on its decade-old promise: to synthesize biomimetic, fully artificial “antibodies.” The example of HSA detection clearly demonstrates their potential in both direct and competitive assay formats—compared with other proteins such as BSA, lysozyme, pepsin, and insulin, with a range of accuracy of 98.4–106.5%. In addition, the LoD and LoQ values recorded were 80 nM and 244 nM, respectively. This is a first step for potential commercialization and may help in overcoming the limitations of using natural antibodies, mainly their high cost and limited ruggedness.

## Supplementary Information

ESM 1 (DOCX 661 kb)
