# Supplementary material for: QCM-based assay designs for human serum albumin
Source: Anal Bioanal Chem. 2021 Dec 23;414(1):731–41. doi: 10.1007/s00216-021-03771-0 (PMC8748353; doi:10.1007/s00216-021-03771-0)

Supplementary material

**QCM-based assay designs for Human Serum Albumin**

Wisnu Arfian A. Sudjarwo^1^, Mathias Thomas Dobler^1^, Peter A. Lieberzeit^1^

^1^Department of Physical Chemistry, University of Vienna, Wahringerstrasse 42, Vienna, Austria

Corresponding author: [Peter.Lieberzeit@univie.ac.at]

**Stern Volmer Plot**

Stern-Volmer plot can reveal affinity binding as the constant, following this equation:

$\frac{I_{0}}{I}=1+K_{q}.\tau_{0}.\left[ Q \right]=1+K_{sv}.\left[ Q \right]$…(1)

where I_0_ and I express steady-state emission intensity of fluorescence without and with nanoparticles as quenchers respectively, Kq is quenching rate coefficient, $\tau_{0}$ states the average of lifetime of protein in the absent of quencher, Ksv represents the Stern-Volmer affinity binding and Q is the concentration of nanoparticles.

**Calculating the maximum expected frequency shift by organic polymer nanoparticle monolayer:**

One can estimate the mass effects expected when exposing the surfaces to the silica nanoparticles based on their sizes and masses using the Sauerbrey equation. For doing so, we need to following data:

Nanoparticle radius: r^MIP^ = 26.5 nm

QCM electrode radius: r^QCM^ = 2 mm

Fundamental frequency f_0_ QCM = 10 MHz = 10^7^ Hz

Density of quartz ρ^SiO2^ = 2.648 g/cm^3^

Shear modulus µ^QCM^ = 2.947 x 10^11^ g.Hz^2^/cm

From this one can calculate both the volume of the average MIP nanoparticle and the area it occupies:

V^MIP^= $\frac{4}{3}\pi x r^{3}= \frac{4}{3}\pi x \left( 26.5 nm \right)^{3}= 77951 {nm}^{3}=7.80 x 10^{-17}{cm}^{3}$

A^MIP^ = $\pi x r^{2}=2206 nm^{2}=2.21 x 10^{-9}mm^{2}$

The area of the QCM electrode is:

A^QCM^ = π x r^2^ = π x (2 mm)^2^ = 12.57 mm^2^

Disregarding hexagonal packing etc., the maximum possible number of nano-MIPs bound to the surface must therefore be below the ration of the two areas:

$$\frac{A QCM}{A MIP}= \frac{12.57 mm^{2}}{2.21 x 10^{-9}mm^{2}}=5.7 x 10^{9} particles$$

If we assume that ρ MIP = ρ H_2_O = 1 g/cm^3^, then the mass of each nanoparticle will be = $7.79 x 10^{-17} g/particle$.

Therefore, the total mass of nanoparticles on electrode will be:

$$n=7.79 x 10^{-17}\frac{g}{particle}x 5.7 x 10^{9} particles=4.44 x 10^{-7}g$$

Based on the Sauerbrey equation:

$$\Delta f=\frac{-2\Delta m{f_{0}}^{2}}{A\sqrt{\rho\mu}}$$

one can calculate:

$$\Delta f=\frac{-2 x 4.44 x 10^{-7}g x (10 x 10^{6}Hz)^{2}}{0.13 cm^{2}\sqrt{2.648\frac{g}{cm^{3}}x 2.947 x 10^{11} g.\frac{Hz^{2}}{cm}}}=-799 Hz$$

Therefore, an ideally densely packed monolayer of the MIP nanoparticles leads to frequency shifts below -800Hz.

**Supplementary Figures:**

**Figure S1** Measuring cell setup


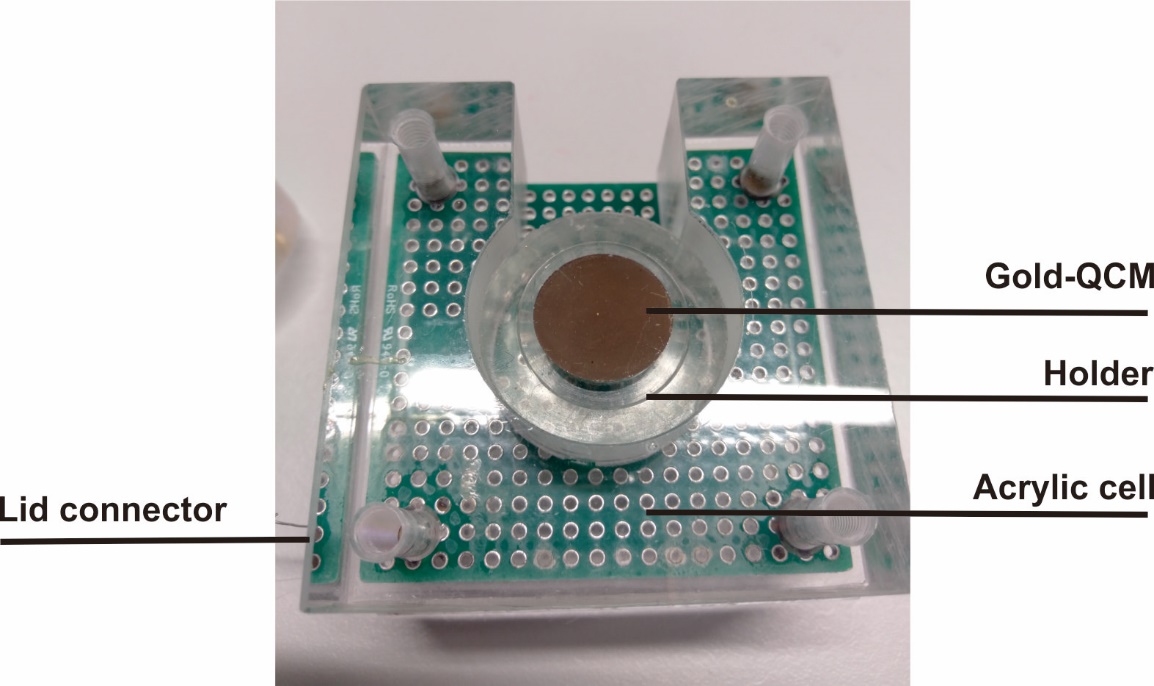


**Figure S2** DLS spectra of nanoparticles. a) Nano-MIPs, b) Nano-NIPs (every nanoparticle batch was measured three times)


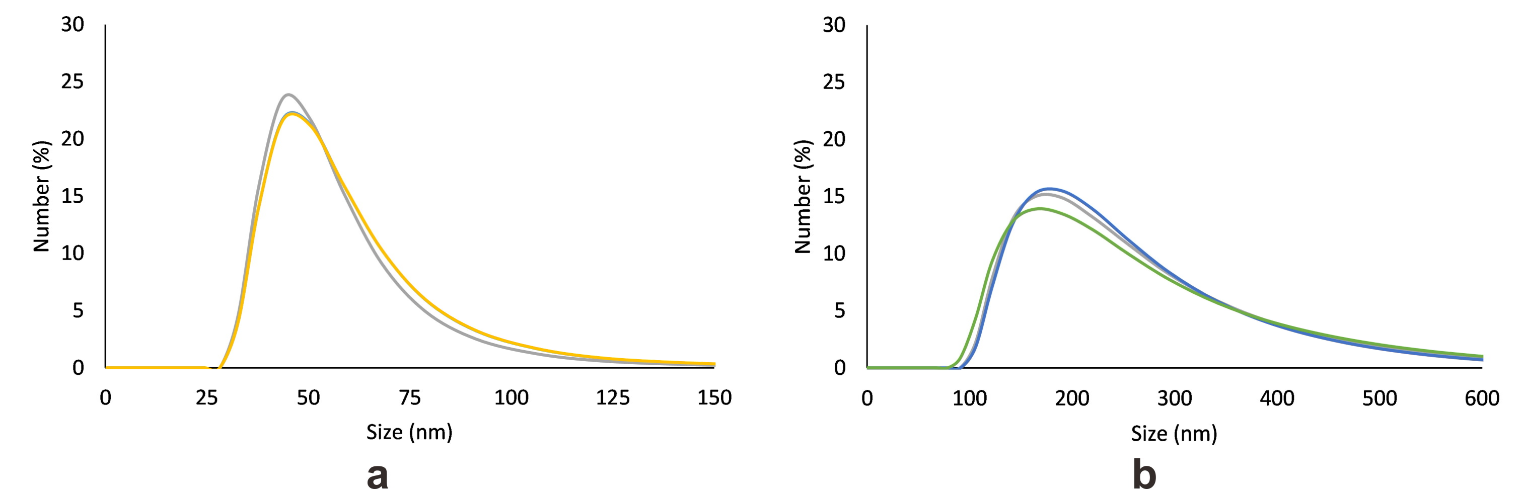


**Figure S3:** Stern-Volmer plots of binding interactions between both nanoparticle types and all selected proteins

**Figure S4:** Single channel QCM data for self-assembled layer to ensure the immobilization of a) Lysozyme, b) Pepsin, and c) BSA


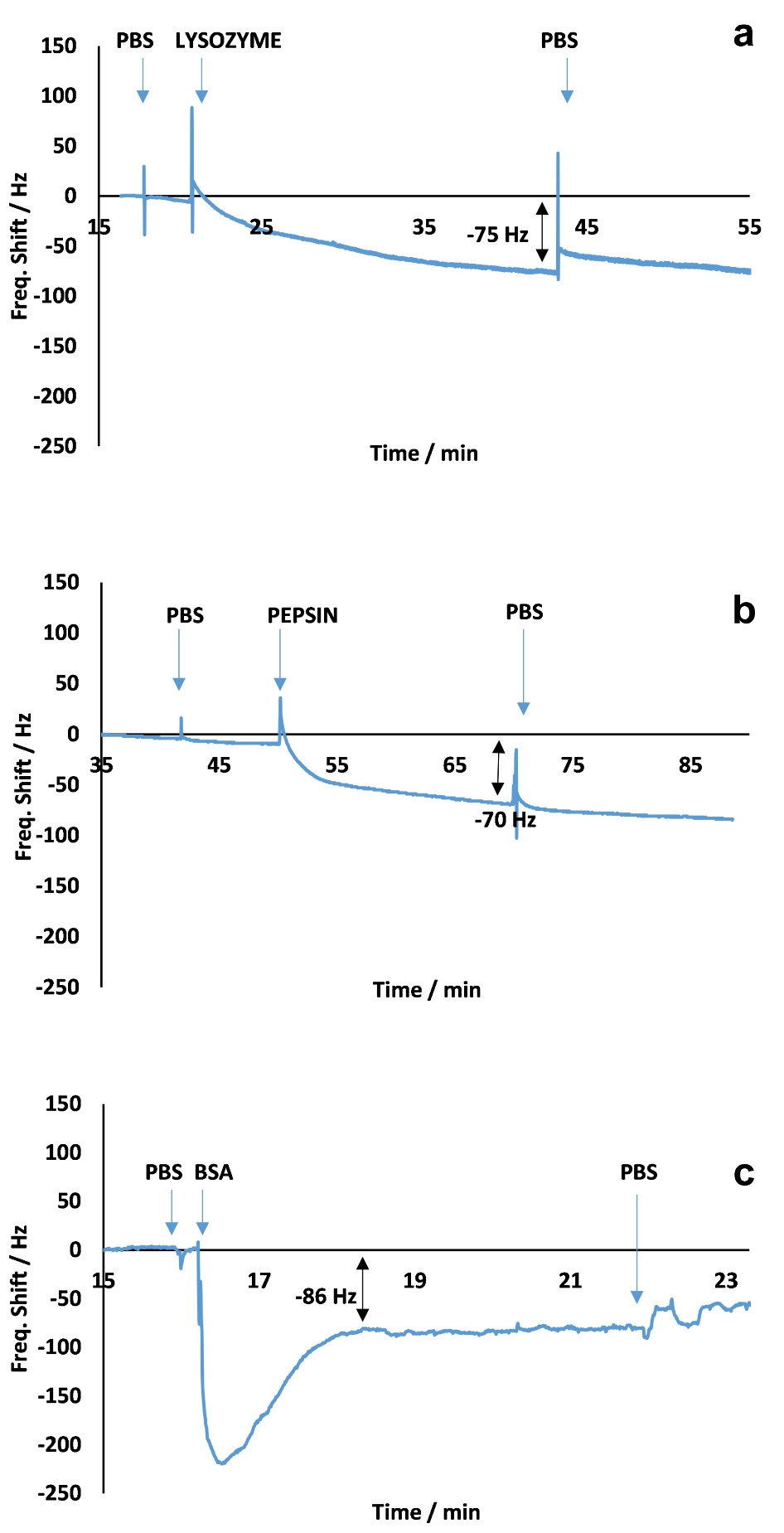


**Figure S5** Binding affinities between nanoparticles and pepsin and lysozyme. a) Interaction nano-NIPs – proteins, b) Interaction of nano-MIPs – proteins


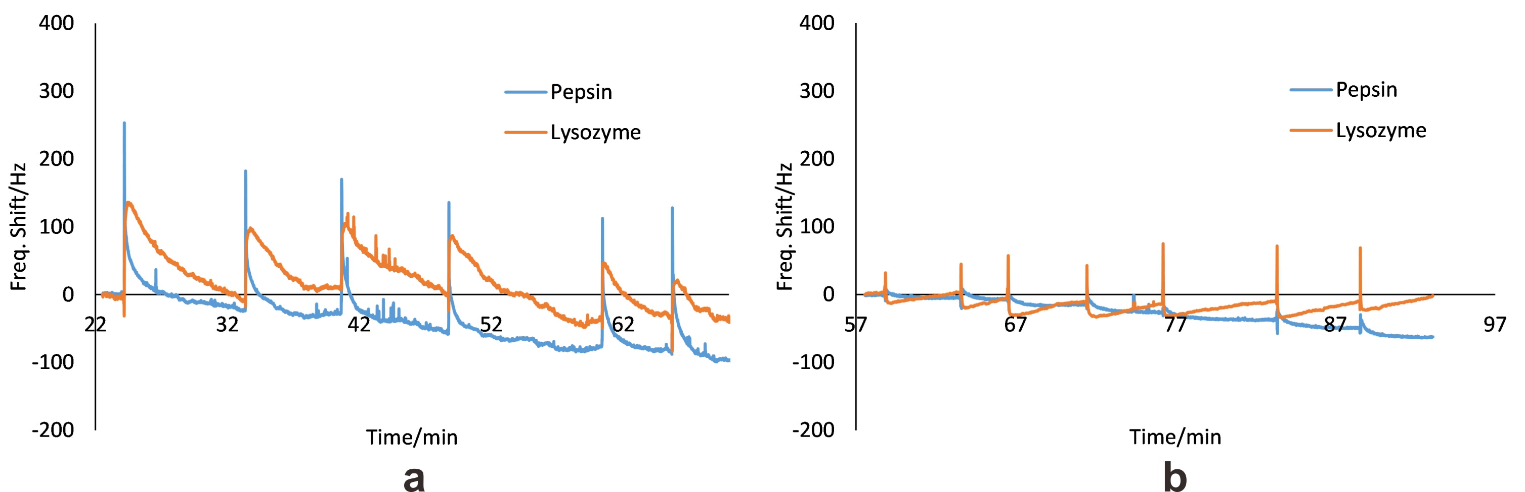

Supplement: Supplementary file 1 — (DOCX 661 kb) [file 216_2021_3771_MOESM1_ESM.docx]
